# CBD Inhibits In Vivo Development of Human Breast Cancer Tumors

**DOI:** 10.3390/ijms241713235

**Published:** 2023-08-26

**Authors:** Lázaro García-Morales, Mónica G. Mendoza-Rodríguez, José Tapia Ramírez, Isaura Meza

**Affiliations:** 1 Department of Molecular Biomedicine, Centro de Investigación y de Estudios Avanzados del Instituto Politécnico Nacional, Avenida Instituto Politécnico Nacional 2508, Ciudad de México 07360, Mexico; lazaro.garcia@cinvestav.mx; 2 Unidad de Investigación en Biomedicina, Facultad de Estudios Superiores-Iztacala (FES-I), Universidad Nacional Autónoma de México (UNAM), Tlalnepantla 54090, Mexico; monica.mendoza@iztacala.unam.mx; 3 Department of Genetics and Molecular Biology, Centro de Investigación y de Estudios Avanzados del Instituto Politécnico Nacional, Avenida Instituto Politécnico Nacional 2508, Ciudad de México 07360, Mexico; jtapia@cinvestav.mx

**Keywords:** CBD-treatment, breast cancer, EMT blockage, tumor resorption

## Abstract

Inflammation is a critical component of cancer development. Previously, we showed in vitro that IL-1β treatment of non-invasive human breast cancer MCF-7 cells promoted their transition to a malignant phenotype (6D cells). This epithelial–mesenchymal transition was reverted by exposure to cannabidiol (CBD). We show in a murine model that subcutaneous inoculation of 6D cells induced formation and development of tumors, the cells of which keep traits of malignancy. These processes were interrupted by administration of CBD under two schemes: therapeutic and prophylactic. In the therapeutic scheme, 6D cells inoculated mice developed tumors that reached a mean volume of 540 mm^3^ at 45 days, while 50% of CBD-treated mice showed gradual resorption of tumors. In the prophylactic scheme, mice were pre-treated for 15 days with CBD before cells inoculation. The tumors formed remained small and were eliminated under continuous CBD treatment in 66% of the animals. Histological and molecular characterization of tumors, from both schemes, revealed that CBD-treated cells decreased the expression of malignancy markers and show traits related with apoptosis. These results confirm that in vivo CBD blocks development of breast cancer tumors formed by cells induced to malignancy by IL-1β, endorsing its therapeutic potential for cancer treatment.

## 1. Introduction

The presence of inflammatory cytokines in the tumor microenvironment has been considered a critical factor for cancer progression [1]. Highly increased serum levels of the inflammatory cytokine IL-1β have been related to the tumor stage in breast cancer patients as an important prognostic marker [2]. Our previous work, utilizing an in vitro model of non-invasive epithelial human breast cancer cells (MCF-7), demonstrated that treatment with the inflammatory cytokine IL-1β transforms these cells to malignancy (thereafter, 6D cells), through an epithelial–mesenchymal transition (EMT) process [3,4]. The EMT was initiated in the MCF-7 cells by activation of a novel IL-1β/IL-1R/β-catenin signaling pathway [4].The transformed 6D cells showed striking changes in the epithelial phenotype, disruption of membrane junctions and dispersion from the monolayer, acquiring high motility. Furthermore, translocation of β-catenin from the membranes to the cell nuclei was observed, followed by upregulation of several genes and proteins such as EGFR, Wip1, ATM, BIRC3 and ∆Np63α, known as malignancy markers of invasive breast cancer cells [5,6,7]. The 6D cells also showed resistance to anticancer drugs doxorubicin and cisplatin [6,7]. These results indicated that the inflammatory cytokine IL-1β by itself activates molecular mechanisms that lead the human breast cancer MCF-7 cells into an aggressive phenotype.

In recent years, research on cancer treatment has focused on developing drugs to increase tumor cell death, reduce tumor volume and block metastasis [8]. Phyto-cannabinoids are chemical compounds with psychoactive and non-psychoactive effects obtained from *Cannabis sativa* [9]. CBD represents the main non-psychoactive compound that is legally approved for use in research and has been frequently used as a palliative in inflammatory diseases and as an inhibitor of cancer progression in patients with different types of cancer [10,11,12]. In our previous in vitro study using 6D cells, the role of CBD was demonstrated to be an inhibitor of the EMT induced by IL-1β, leading to downregulated expression of proteins and features identified as malignant markers of the EMT processes. As a result, the transformation of the cells to malignancy was hindered and cells reverted to an epithelial phenotype [13].

It has been emphasized that the complexity of the tumor microenvironment is affected not only by the heterogeneity of cancer cells, but also by the variable composition of their surrounding stromal cells [14,15]. We therefore implemented an in vivo model to study the antitumorigenic properties of CBD, following two schemes of administration: therapeutic (TS) and prophylactic (PS). In both schemes, solid tumors were induced in nu/nu mice by injection of 6D cells. Developed tumors were then analyzed to evaluate if CBD could block the transition to malignancy induced by IL-1β. It was found that CBD significantly inhibited development of tumors and, in several cases, induced tumor resorption. In CBD-untreated tumors, the expression of malignant markers in the tumor cells was retained. In contrast, cells from tumors treated with CBD showed downregulation of the expression of malignant traits. These results validated the anti-inflammatory activity of CBD and support its use not only as a palliative, but also as a potential therapeutic anticancer drug.

## 2. Results

### 2.1. CBD Antitumor Effect under Therapeutic Conditions

**Figure 1:**
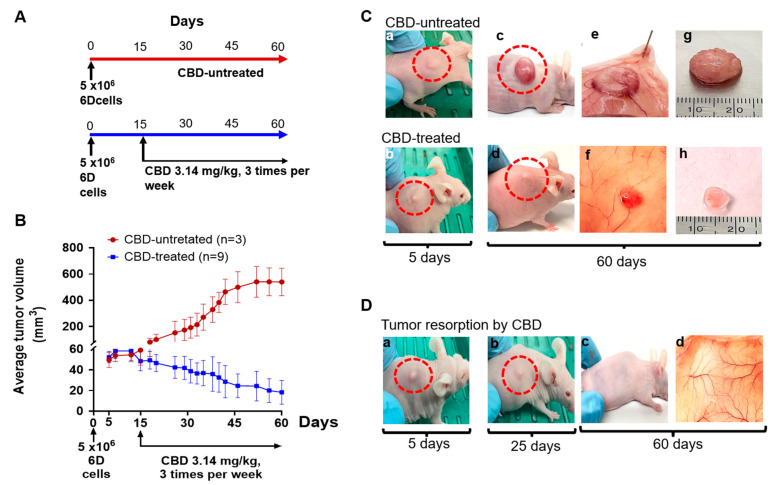
CBD effect on the size of tumors induced in mice by inoculation of 6D cells. (**A**) Therapeutic scheme used for the administration of CBD in mice inoculated with 6D cells. (**B**) Tumor volume from day 5 to day 60 after the initial injection of 6D cells in CBD-untreated and CBD-treated mice. (**Ca**,**Cb**) Representative images of tumors developed at day 5 from the group of CBD-untreated mice and the CBD-treated mice group. (**Cc**,**Cd**) Images of tumors developed till 60 days. (**Ce**,**Cf**) Vascularization of tumors and the surrounding tissue. (**Cg**,**Ch**) Tumors removed from the mice at day 60. (**D**) Representative images of tumor resorption induced by CBD. (**Da**) Tumor in a mouse after 5 days of injection of 6D cells. (**Db**,**Dc**) Significant reduction of growth is shown in the mouse at day 25 and complete resorption at day 60. (**Dd**) Vascularization area in the site of the reabsorbed tumor.

As a first approach, we evaluated the capacity of the malignant 6D cells to induce tumors in vivo. For this, a group of nu/nu female mice were inoculated with 6D cells, as indicated in Figure 1A and the development of tumors was followed for 60 days, when the animals were euthanized. Another group was treated with CBD (3.14 mg/kg) 15 days after the injection of 6D cells and from this time CBD was administered three times per week up to day 60 (Figure 1A, TS conditions). 0.01% coconut oil dissolved in BSA was used as the vehicle control in CBD-untreated mice.

Figure 1B shows that, 5 days after the inoculation of the mice with 6D cells, the tumors that developed at the injection site reached a mean volume of 50 mm^3^ and grew up to a mean volume of 540 mm^3^ by the end of the experiment (day 60). These results showed that 6D cells have the capacity to induce in vivo the formation of solid tumors. The effect of CBD on the tumor development was evaluated 15 days after the first inoculation with the 6D cells under TS conditions. The graph in Figure 1B shows a slight volumetric reduction of the tumors 5 days after the first CBD administration. By day 30, the CBD-treated tumors had reduced by 50% of their volume compared to the mean volume of the group of tumors not-treated with CBD, reaching a mean volume of 80 mm^3^. After 45 days of CBD continuous peritumoral administration, the tumors were reduced to an average volume of 18 mm^3^ and in 33% of this group of mice total reabsorption had taken place. These results showed that treatment with CBD under TS conditions blocked tumor development and promoted elimination.

Figure 1(Ca,Cb) show representative images of a group of mice with induced tumors after five days of inoculation with 6D cells in CBD-untreated and CBD-treated mice. Figure 1(Cc,Cd) show in the same mice the tumors at 60 days, which grew as a compact cell mass with a volume of 546 mm^3^. Figure 1(Ce) shows the striking increase in the peritumoral vascularization present in the subcutaneous tissue of a representative CBD-untreated mouse. Figure 1(Cf) shows a smaller tumor and normal peritumoral vascularization in the tissue of a representative CBD-treated mouse. Figure 1(Cg,Ch) show the significant volume difference between the dissected tumors from the CBD-untreated mice and the CBD-treated mice (560 mm^3^ vs. 30 mm^3^, respectively). Throughout the experiments, all mice whether CBD-treated or CBD-untreated, increased their body weight by the same rate.

Figure 1D shows the representative images of a mouse from the group of CBD-treated mice, where the complete reabsorption of the tumor was observed. In this mouse, at day 5 (Figure 1(Da)), the incipient tumor formed was similar to the tumors formed at the same time as in the group of CBD-untreated mice, as shown in Figure 1(Ca). However, on day 25, this CBD-treated tumor was reduced by 45% in size (Figure 1(Db)) and was totally reabsorbed at day 60 (Figure 1(Dc)). After dissection of the mouse, no tumor remnants were visible at the site of the tumor and the tissue had normal vascularization (Figure 1(Dd)). These results, together, with those previously reported in vitro, support the selective antitumorigenic effect of CBD when applied in advance of the tumor cells.

### 2.2. CBD Treatment of Mice under Prophylactic Conditions

The results above showed that CBD inhibits tumor progression when administered 15 days after inoculation with the malignant 6D cells. However, some reports using other cancer cells models attribute to CBD a time-dependent activity [16,17,18]. Here, we investigated in our in vivo model this property of CBD, under PS conditions.

**Figure 2:**
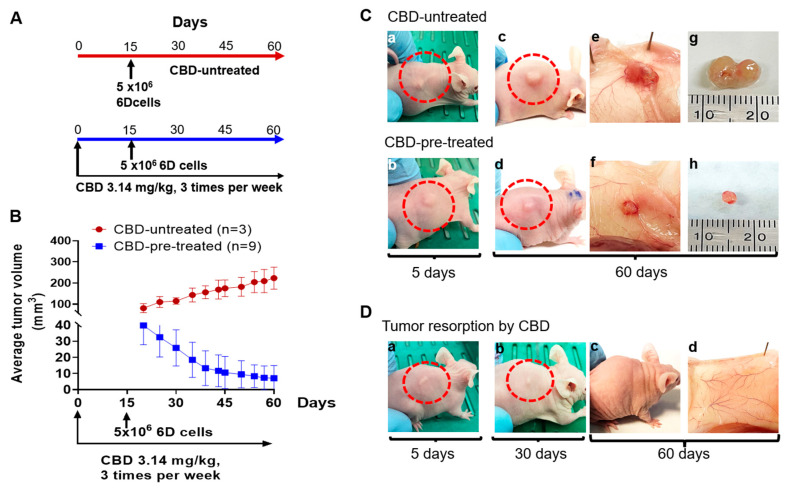
CBD as a pre-treatment of tumor formation. (**A**) Prophylactic scheme used for the administration of CBD in mice inoculated with 6D cells. (**B**) Tumor volume from day 20 to day 60 after the initial injection of 6D cells. (**Ca**,**Cb**) Representative images of tumors developed at day 5 from the group of CBD-untreated mice and of the group of CBD-pre-treated mice. (**Cc**,**Cd**) Images of the tumors developed till 60 days. (**Ce**,**Cf**) Vascularization of the tumors and the surrounding tissue. (**Cg**,**Ch**) Size of the tumors removed from the mice at day 60. (**D**) Representative images of tumor resorption induced by CBD pre-treatment. (**Da**) Tumor in a mouse after 5 days of injection of 6D cells. (**Db**,**Dc**) Significant reduction of growth is shown in the mouse at day 30 and complete resorption at day 60. (**Dd**) Vascularization area in the site of the reabsorbed tumor.

The graph in Figure 2A shows that mice pre-treated with CBD for 15 days and then injected with 6D cells developed, in 5 days, tumors that were 50% smaller than those induced in mice not pre-treated with CBD. After 60 days of continuous peritumoral CBD administration, the tumors were reduced to an average volume of only 7.1 mm^3^, while in CBD-untreated mice the tumors reached an average volume of 223 mm^3^. Furthermore, under these PS conditions, tumor resorption was observed in 66% of the animals at the end of the experiment (60 days). These results showed that pre-treatment of mice with CBD before inoculation with the 6D cells delays tumor development and favors their resorption.

Figure 2(Ba,Bb) show representative images of mice in which the tumor developed five days after inoculation with 6D cells in CBD-untreated or in CBD-pre-treated mice. The development of tumors in the CBD-pre-treated mice was low compared with the development in the CBD-untreated mice. The vascularization around the solid cell mass was also limited (Figure 2(Bc–Bf)). Figure 2C shows that, during PS conditions with CBD, the mice increased their body weight by 34%. After 60 days of receiving CBD treatment the tumors had almost disappeared, and the surrounding tissue recovered its normal vascularization (Figure 2(Dd)). These findings, along with the ones mentioned above, in the TS, showed that CBD may have both therapeutic and prophylactic effects.

### 2.3. CBD Effects on Tumor Histology

**Figure 3:**
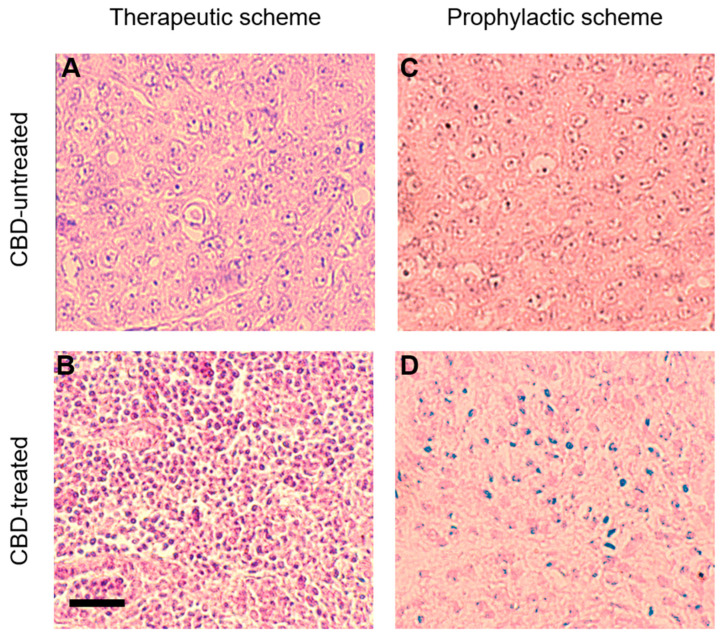
Sections of CBD-untreated and CBD-treated tumors developed in mice in both schemes of administration. (**A**,**B**) Sections of CBD-untreated and CBD-treated tumors in the TS. (**C**,**D**) Sections of CBD-untreated and CBD-pre-treated tumors in the PS. Tissues were stained with H&E, bar = 50 µm.

As reduction in the size of the tumors was observed under CBD administration for a long period (25 to 30 days), the histology of CBD-treated tumors was analyzed and compared with the histology of tumors not-treated with CBD. Figure 3A,C show the hematoxylin and eosin (H&E) sections of CBD-untreated tumors from the TS and the PS, conditions, respectively. The tumor cells in either condition showed significant heterogeneity in size, a compact distribution mixed with surrounding stromal cells and lack of the regular tubular arrangement reported in normal breast tissue [19]. These cells also have nuclear pleomorphism, exhibiting frequent mitotic stages, indicating that 6D cells are actively replicating in the tumors.

In contrast, tumors from CBD-treated mice under the TS showed a striking decrease in the size of the cells and predominant pyknotic nuclei suggestive of tumor cell death (Figure 3B). In the PS, the tumor cells also reduced their size and their nuclei showed condensed and fragmented chromatin (Figure 3D). In both treatment conditions, CBD decreased the expression of the Vascular Endothelial Growth Factor (VEGF), to 22% in tumors treated under TS, and to 8% in tumors from PS, compared with the 100% relative value of the CBD-untreated tumors used as control (Supplementary Figure S1). These results suggest that CBD could be inducing the elimination of the 6D breast cancer cells.

### 2.4. CBD Modifies the Expression of Tumor Development Markers Ki67, Bcl2 and P53

Results above showed that in mice CBD also promotes tumor resorption as well as histological changes in the cells that show a decline of the cellular structure leading to cell death. Therefore, to determine if the resorption and elimination of the tumors could possibly be induced by an apoptosis process, the expression of apoptotic-markers Bcl2 and P53 was evaluated. At the same time, cell proliferation was evaluated by the expression of protein Ki67, a protein expressed during cellular division.

**Figure 4:**
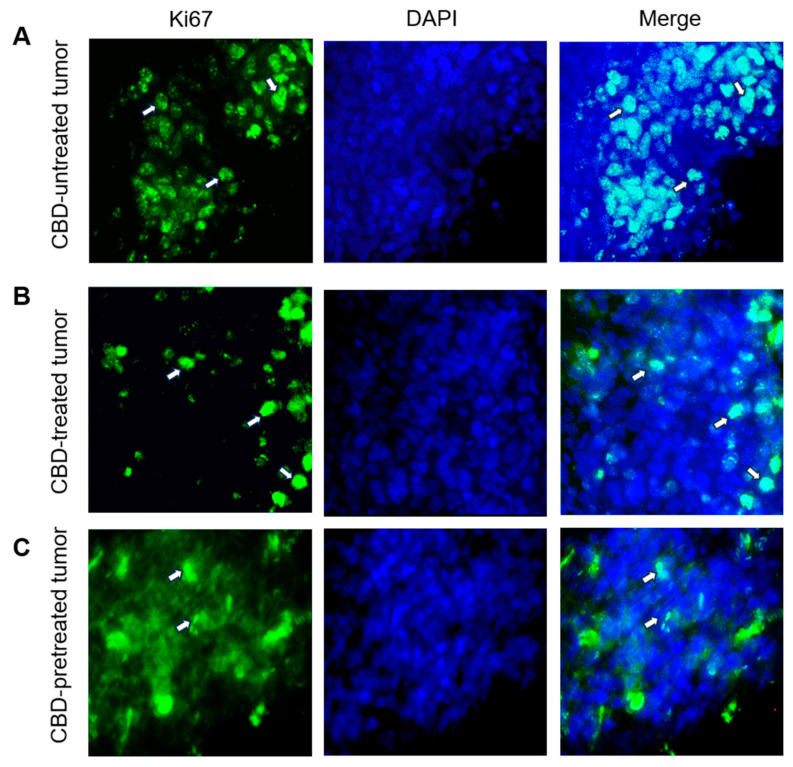
Immunofluorescence localization of the nuclear protein Ki67 in tumor sections. (**A**) Expression of Ki67 in the cell nuclei of CBD-untreated tumor cells stained with the specific antibody to Ki67 (arrows). (**B**) Expression of Ki67 in the nuclei of cells from tumors that were CBD-treated under TS (arrows). (**C**) Expression of Ki67 in the nuclei of tumor cells from CBD-pretreated mice under PS (arrows). Nuclei were stained with DAPI. Representative images obtained from three different tumors. Bar = 50 µm.

Figure 4A shows the immunolocalization of the protein Ki67 in the nuclei of the cells. The percentage of positive stained cells was determined after analyzing 300 cells in three different fields. In CBD-untreated cells, 65% expressed this marker. In contrast, in cells coming from CBD-treated tumors, this nuclear marker was only present in 15% of the counted cells from tumors treated under TS condition and 10% in cells from the PS condition. Additionally, in some nuclei of CBD-treated tumor cells, in both conditions, condensation of the chromatin was visualized.

**Figure 5:**
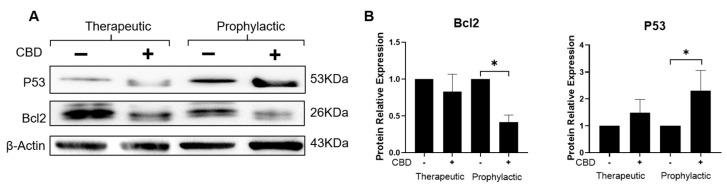
CBD dysregulates the expression of apoptosis-regulatory proteins P53 and Bcl2. (**A**) Representative western blot of proteins P53 and Bcl2 from 60-day tumors from mice treated or not treated with CBD under TS and PS conditions. (**B**) Densitometric analysis of the expression levels of Bcl2 and P53 proteins. The values were normalized to actin and expressed relative to those in CBD-untreated tumors. Data represent the average of three different tumors. Asterisks indicate *p* < 0.05.

The possibility of expression of apoptosis-related markers Bcl2 and P53 was evaluated in tumor cell extracts by western blots using specific antibodies for these proteins (Figure 5A). Figure 5B shows that CBD decreased the expression of the anti-apoptotic protein Bcl2 to 83% in tumors treated under TS, and to 42% in tumors from PS compared with the 100% relative value of the CBD-untreated tumors used as control. In contrast, the expression of the pro-apoptotic protein P53 was increased 1.5-fold in CBD-treated tumors under TS conditions and 2.3-fold in tumors from CBD-pre-treated mice under PS conditions. These results and the decreased expression of the Ki67 protein in cells treated with CBD suggest that CBD is an inhibitor of tumor development through decrease of cell proliferation rate by an apoptosis-like process.

### 2.5. CBD Modifies Expression of Effectors Participating in the IL-1β/IL-1R/β-Catenin Pathway

As shown above, 6D cells injection in nu/nu mice induced the formation of tumors whose development was inhibited by administration of CBD. The tumor cells showed morphological and molecular features indicating that they kept the expression of malignancy markers acquired by their treatment with IL-1β and its activation of the IL-1β/IL-1RI/β-catenin pathway. Therefore, we analyzed the CBD effects found in vivo to see if they could be associated with the inhibition of the IL-1β signaling, as previously reported in vitro [13].

**Figure 6:**
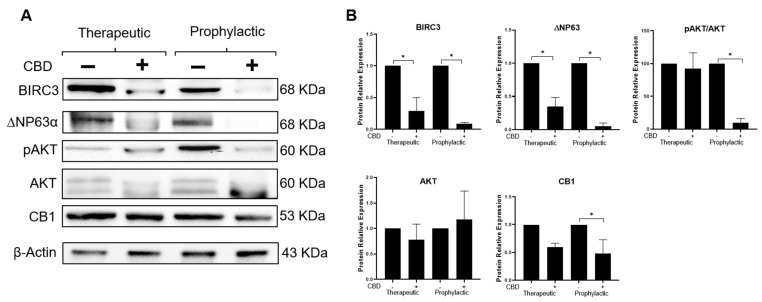
CBD dysregulates the expression of effectors of the IL−1β/IL-IR/β-catenin signaling pathway in tumors. (**A**) Representative western blot of the proteins BIRC3, ∆NP63α isoform, pAKT (Ser473), AKT and CB1 isolated from 60-day tumor cells that were treated with CBD (TS) or from mice that were pretreated with CBD (PS). (**B**) Densitometric analysis of protein expression; the values were normalized to actin and reported relative to those in cells of CBD-untreated tumors. Data represent the average of three different tumors. Asterisks indicate *p* < 0.05.

It was previously demonstrated in vitro that high expression levels of the CB1 receptor of CBD is necessary for its internalization and its activity as an anti-inflammatory agent blocking the IL-1β signaling in the 6D cells [13]. It is shown now that, in vivo, CBD treatment of tumors reduced the expression of CB1 to 60% in the cells under the TS conditions (Figure 6A,B). The reduction of CB1 levels was more evident (to 48%) in tumor cell extracts from animals pre-treated with CBD under PS conditions, indicating that this cannabinoid promotes in the tumor cells the loss of features acquired by exposure to IL-1β.

To test the blocking effect of CBD on the IL-1β/IL-1RI/β-catenin pathway, evaluation of the synthesis and activation of AKT by phosphorylation at Ser473 (crucial cross-talk between IL-1β pathway and CBD) was performed in vivo (Figure 6B). It was found that CBD reduced by 8% the activation of AKT when this cannabinoid was administered in mice under TS conditions, while under PS conditions AKT phosphorylation was reduced by 90%. As significant change in the expression of total AKT was not found, CBD is only decreasing the phosphorylation of AKT.

To support the above results, the expression of two downstream effectors, participating in the IL-1β/IL-1RI/β-catenin pathway by increasing resistance of 6D cells to anti-cancer drugs, was analyzed [6,7]. Figure 6B shows that in vivo CBD treatment of the tumors caused downregulation of BIRC3 and ΔNP63α expression. Under therapeutic conditions, BIRC3 was reduced by 70% and ΔNP63α by 66% and, under prophylactic conditions, BIRC3 was reduced by 90% and ΔNP63α by 94%. These data also support the participation of CBD in the blockage of the IL-1β/IL-1RI/β-catenin pathway leading to inhibition of tumor development.

## 3. Discussion

Several reports in the cancer literature indicate that an inflammatory microenvironment is a critical factor for cancer progression and a potential target for therapy [1,18,20,21]. In our in vitro studies, using as a model non-invasive human breast cancer MCF-7 cells, we demonstrated that treatment with the inflammatory cytokine IL-1β produces alterations in the regulatory mechanisms of growth and differentiation, which, through an EMT process, transformed the cells to an aggressive phenotype with resistance to anticancer drugs [4,6,7].

In vitro models are very useful in cancer studies, as specific cell types can be chosen and exposed to strictly controlled experimental conditions. However, they have only a limited ability to predict in vivo behavior of the cancer cells in an organism [22,23]. The murine model used in this work allowed us to study the formation and resorption of tumors obtained from injected malignant human breast cancer 6D cells and to monitor the effects of CBD treatment on the time course of these processes.

In this study, we demonstrated the capacity of cultured malignant 6D cells to form solid tumors in vivo without necessary addition of several components, such as cytokines, extracellular matrix (matrigel^®^) or estrogens, in contrast with data reported by other authors for MCF-7 breast cancer cells [24,25]. Characterization of the induced tumors, using histological and molecular methods, showed that tumor cells retained malignant features, such as increased replication and expression of malignant markers induced by exposure to IL-1β, thus validating our in vitro data and showing that these effects were not solely artefacts of cultivation. This observation is interesting, as isolated and cultivated primary cells typically differ from corresponding cell types in an organism [26].

Previously, in our in vitro model, we showed that CBD at a concentration not higher than 10 µM, blocked the expression of malignant traits in the 6D cells, by the inhibition of IL-1β signaling [13]. Based on these data, we established 3.14 mg/kg of CBD as the dose to be used in vivo. Other authors using greater than 10 mg/kg doses reported deleterious effects in animals treated with CBD, such as toxicity to embryo–fetal development, neurotoxicity, hepatocellular lesions, reduction in spermatogenesis, organ weight alterations and changes in the male reproductive system [27]. With our established conditions, we could study the inhibitory effect of CBD on tumor growth and survival, i.e., in conditions more resembling cancer pathology, making CBD a more reliable anticancer drug.

Here, using two schemes of administration, therapeutic (TS) and prophylactic (PS), the time-dependent effect of CBD was analyzed. Under TS, CBD was not administered till 15 days after the injection of 6D cells but was then administered to the end of the experiment. In PS, CBD was administered 15 days before the injection of 6D cells, and the concentration of peritumoral CBD was maintained to the end of the experiment. The results obtained with the PS conditions showed a strong negative effect of CBD which caused a large decrease in the size of the tumors, as well as downregulation of the expression of the malignancy markers, showing that CBD has a preventive effect on tumor development. Although, in the TS conditions, similar anti-tumorigenic properties were shown by CBD, our results suggest that the efficacy of the treatment will be greater when the tumor is detected in an early stage of development. Our two protocols showed that CBD both inhibits the formation of a tumor and hastens its reduction, raising the question of what happens to the tumor cells: do they modulate, or do they die? Previously, in the in vitro model it was observed that decreased expression of ΔNP63α and BIRC3 protein effectors downstream in the IL-1β/IL-1RI/β-catenin pathway was associated with an apoptosis process, diminishing cell survival [6,7]. Now, in the in vivo model, we confirmed that CBD downregulates the expression of these proteins as well as the levels of Bcl2, favoring overexpression of P53. These proteins could be correlated with a decrease of cell survival. It has been shown that CBD can induce a time-dependent killing effect on tumor cells [16,28], and now our in vivo model provides more reliable knowledge of the tumor progression in which CBD could be a modulator of the tumor micro-environment.

In conclusion, the present study shows that CBD, properly administered, can effectively block development of human breast cancer tumors in vivo, without causing adverse effects, by regulating in the tumor cells the expression of malignant traits and bearing characteristics of a possible route via apoptosis, both favorable attributes for an anticancer drug.

## 4. Materials and Methods

### 4.1. Reagents

The RH-Oil5 ™ containing 23.36 mg/mL of purified CBD, dissolved in coconut oil, was acquired from (HempMeds™, Monterrey, NL, Mexico). A 1000 μM stock solution was prepared by dilution in DMSO (Sigma-Aldrich, St. Louis, MO, USA). Aliquots from this stock were diluted in 5% BSA solution to obtain a CBD concentration with which to inject mice. Coconut oil (doTERRA™, Pleasant Grove, UT, USA) 0.01% final concentration in BSA was used as the vehicle in CBD-untreated mice.

### 4.2. Primary Antibodies

Specific antibodies used were: anti-ΔNp63α, anti-CB1 and anti-cIAP2/BIRC3 (GeneTex, Irvine, CA, USA); anti-AKT and anti-Phospho-AKT-Ser473 (Cell Signaling Technology, Danvers, MA, USA); anti-β-catenin (Thermo Scientific, Waltham, MA, USA); anti-P53 (Cell Signaling Technology, Danvers, MA, USA); anti-Bcl-2 (Abclonal, Woburn, MA, USA) and anti-Ki67 (Santa Cruz Biotechnology INC., Santa Cruz, CA, USA).

### 4.3. Cell Culture

The 6D cells, clones selected from MCF-7 non-invasive human breast cancer cells, that were highly responsive to IL-1β stimulus and transformation to malignancy by the IL-1β-induced EMT [3,4], were used as a cellular model. Cell culture and IL-1β stimulus were performed as previously described [13].

### 4.4. Animal Model

All animal experiments were performed in accordance with the International Official Regulations and guidelines approved by the UPEAL/CINVESTAV Animal Experimentation Committee (Protocol Number E-0033-19). Animals were housed in the animal facility of CINVESTAV under standard conditions. Efforts to replace, reduce and refine the use of laboratory animals were considered. To avoid irrelevant suffering to treated mice, euthanasia was performed as soon as the studied times were reached or when the animals achieved a tumor diameter higher than 1.5 cm or weight loss of more than 25%. To minimize suffering and mice distress, environmental enrichment of carton-board fun tunnels and chewing blocks were provided.

Experiments were performed with athymic female nude mice (3/4-week-old nu/nu, Charles River Laboratories, Wilmington, DE, USA). Tumors were generated by subcutaneous injection of 5 × 10^6^ 6D cells resuspended in 200 μL of saline solution, following two schemes of administration: (1) prophylactic (PS) and (2) therapeutic (TS). In the PS protocol, treatment with CBD was initiated 15 days before the 6D cell inoculation. Treatment with CBD was initiated after 15 days in the TS protocol. In both protocols, 3.14 mg/kg of CBD was injected three times per week up to 60 days [13]. A group of animals used as controls in each protocol did not receive CBD treatment and were injected with the CBD vehicle (Coconut oil). Up until the completion of the experiment (60 days), measurements of the animal weight and tumor size (reported as volume) were made twice a week. Tumor volume was calculated using the formula V = (L × W^2^)/2 where V is tumor volume, W is tumor width, and L is tumor length. In both schemes, tumor volume was measured after the first appearance (5 days after cell inoculation). At day 60, mice were euthanized, and tumors removed were prepared for immunofluorescence, H&E stain and to obtain cell extracts.

### 4.5. SDS-PAGE and Western Blotting

Tumors were lysed mechanically using an Eppendorf pellet pestle in the presence of 200 µL of 1× RIPA buffer supplemented with Complete™ Protease Inhibitor Cocktail (Roche Applied Science, Mannheim, Germany). Protein concentrations were determined by the BCA method (Pierce™ BCA Protein Assay Kit). Thirty micrograms of protein were loaded per lane and separated by SDS-PAGE in 10 and 12% polyacrylamide gels, blotted onto nitrocellulose membranes and blocked with Blotting-Grade Blocker™ (Bio-Rad, Hercules, CA, USA). Membranes were exposed to the anti-human antibodies listed above. The anti-β-actin monoclonal antibody, kindly donated by JM Hernández (CINVESTAV-IPN), was used to detect actin as a loading control. The HRP-tagged secondary antibodies were anti-rabbit or anti-mouse (1:5000) (Jackson Immuno Research, West Grove, PA, USA). Chemiluminescent detection was done with Immobilon™ and recorded on a ChemiDoc™ imaging device (Bio-Rad Laboratories, Hercules, CA, USA) for densitometric analyses with Image Lab software (v 6.0, Bio-Rad Laboratories, Hercules, CA, USA). Western blots were obtained after three independent experiments.

### 4.6. Histologic Analysis and Immunofluorescence Staining

Tumors induced by inoculation of 6D cells were collected from mice treated or untreated with CBD and fixed in 4% PFA, then embedded in paraffin for posterior 4-mm cross-sectioning. The sections were deparaffinized in xylene and then rehydrated with graded alcohols and processed as reported previously [29]. Then, the tissue sections were stained with hematoxylin and eosin (H&E; for pathologic evaluation) or immunofluorescence staining for Ki67 detection. Briefly, the tissues were incubated overnight at 4 °C with the respective primary antibody, Ki67 (Santa Cruz Biotechnology INC., Santa Cruz, CA, USA), diluted (1: 150) in PBS and anti-rabbit-ALEXA 488 at 1:100 dilution for 1 h at RT. Nuclei were stained with DAPI for 2 min. Coverslips were mounted with Fluoro-Gel™ (Electron Microscopy Sciences, Hatfield, PA, USA). Cells were observed in an Olympus I50X epifluorescence inverted microscope. Image-Pro PlusTM software (v. 3.0, Media Cybernetics, Rockville, MD, USA) was used to analyze microscope images taken with an Olympus DP72 digital camera.

### 4.7. Statistical Analysis

Data are presented as mean ± SD. They represent at least three independent determinations (biological replicates) and in all cases *p*-Values ≤ 0.05 were considered significant. The v 8.0 of GraphPad Software (La Jolla, CA, USA) was used for statistical analysis. Multiple comparisons were done using 2-way ANOVA and Dunnett’s multiple comparisons tests.

## Data Availability

Data sharing not applicable. No new data were created or analyzed in this study.

## Supplementary Materials

The supporting information can be downloaded at: https://www.mdpi.com/article/10.3390/ijms241713235/s1.
