# Supplementary material for: CBD Inhibits In Vivo Development of Human Breast Cancer Tumors
Source: Int J Mol Sci. 2023 Aug 26;24(17):13235. doi: 10.3390/ijms241713235 (PMC10488207; doi:10.3390/ijms241713235)
Supplement: Supplementary file 1 [file ijms-24-13235-s001.zip › ijms-2555108-supplementary.pdf]

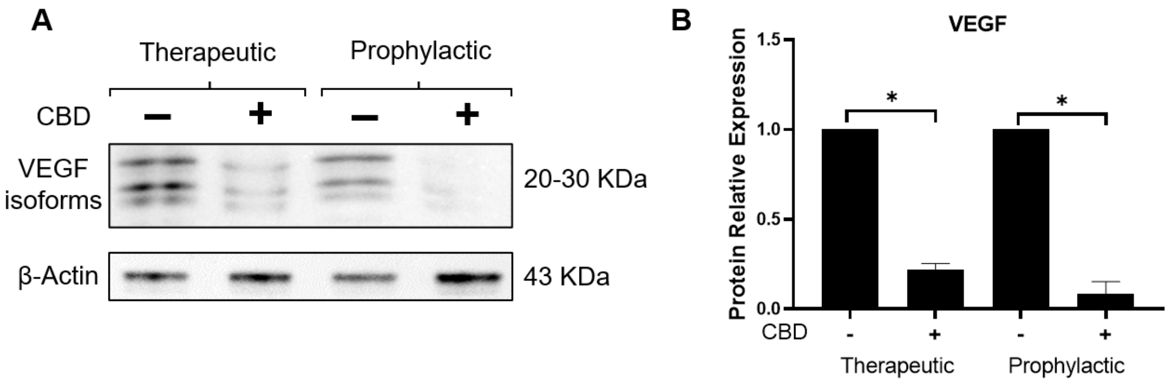

Supplementary figure S1. CBD decreased the expression of VEGF protein in 6D cell tumors. (A) Representative western blot showing the isoforms 121, 165 and 189 of the protein VEGF (Vascular Endothelial Growth Factor) detected by the anti-VEGF (A-20) antibody (Santa Cruz Biotechnology INC., CA, USA). Sixty-day tumors from mice that were treated with CBD or CBD-untreated under TS and PS conditions. **(B)** Densitometric analysis of the expression levels of VEGF. The values were normalized to actin and expressed relative to those in CBD-untreated tumors. Data represent the average of three different tumors. Asterisks indicate $p < 0.05$.
